# Evaluation of Decay Kinetics of Black Elderberry Antioxidants from Fruits and Flowers

**DOI:** 10.3390/antiox13070804

**Published:** 2024-07-02

**Authors:** Iwona Golonka, Andrzej Dryś, Katarzyna Podgórska, Joanna Polewska, Witold Musiał

**Affiliations:** Department of Physical Chemistry and Biophysics, Wroclaw Medical University, Borowska 211A, 50-556 Wrocław, Poland; iwona.golonka@umw.edu.pl (I.G.); katpod2@wp.pl (K.P.);

**Keywords:** *Sambucus nigra*, free radical scavenging, DPPH and Glv radicals

## Abstract

The health-promoting properties of black elderberry are related to its high content of polyphenols (natural antioxidants), which eliminate free radicals and prevent the formation of oxidative stress responsible for many diseases. The aim of this work was to determine, the anti-radical effect of *Sambucus nigra* infusions based on the reaction with 2,2-diphenyl-1-picrylhydrazyl (DPPH) and galvinoxyl (Glv) radicals and to determine the function describing the disappearance curves of these radicals. The antioxidant properties of infusions obtained from the flowers and fruits of this plant were tested using the modified Brand-Williams method using DPPH and Glv radicals. Higher antioxidant activity towards both the DPPH and Glv radicals was found in flowers compared to fruits. In addition, it was found that the process of quenching radicals in the reaction with *Sambucus nigra* infusions proceeds in accordance with the assumptions of second-order reaction kinetics. The infusion obtained from flowers quenched radicals faster than fruit infusions. The applied second-order kinetics equation may enable estimation of antioxidants levels in natural sources of radicals.

## 1. Introduction

Elderberry (*Sambucus nigra* L.) belongs to the elderberry family borers (*Caprofoliaceae*). The composition of individual morphological parts of plants depends on many factors, e.g., variety and environmental conditions. In the case of fruits, their degree of ripeness is important, while in the case of flowers the flowering period is similarly relevant. Variation in the composition of various morphological parts of elderberry is observed primarily in the content of bioactive compounds and antioxidant properties related to the content of these ingredients [1]. The flowers of *S. nigra*, which are also a pharmaceutical raw material, contain even greater amounts of phenolic compounds (rutin, isoquercitrin, and astragalin, all bioactive polyphenols) than the fruits or leaves of this species. They are often standardized to contain at least 0.8% total flavonoids on an isoquercitrin basis [2]. Flavonoids contained in plant materials take part in oxidation and reduction processes inside and outside cells [3,4]. The antioxidant power of flavonoids is based on their ability to interact with free radicals that initiate oxidation reactions or that are formed during chain reactions as well as to inhibit oxidation processes, which reduces activity oxidase enzymes. They can also form complexes with transition metal ions, which are catalysts for oxidation reactions [5,6,7]. The phenolic profile of commercial fruit and flower products of *Sambucus nigra* shows both phenolic acids (caffeic, chlorogenic, p-coumaric acid, ferulic acid, gallic acid, and syringic acid) and flavonols (quercetin, kaempferol, myricetin, and rutin) [8]. Nature has produced many polymers created by combining different monomers. One of these is cellulose (C_6_H_10_O_5_)_n_, a polysaccharide composed of glucose residues connected in long unbranched chains by 1,4-glycosidic bonds of β configuration. The total sugar content in elderberries may range from 68.5 to 104.2 g/kg [9,10,11]. For the Haschberg and Rubini extract, the range of glucose concentration was set from 33.33 to 50.23 g/kg of fresh weight (FW) and the range from 33.99 to 52.25 g/kg for fructose. Another example would be the Sabugueiro and Bastardeira varieties harvested over three consecutive years, which showed glucose levels ranging from 42.7 to 104.9 g/kg FW, fructose levels ranging from 34.9 to 63.4 g/kg FW, and sucrose in a range from 0.6 to 4.8 g/kg FW [12,13]. Literature data shows that the main sugars present in elderberry are glucose and fructose, followed by sucrose, which occurs in much smaller amounts. In the case of elderberry flower extracts the trend appears to be the opposite, as higher sucrose content has been observed, followed by fructose and glucose [14]. The fruits can be considered a good source of fiber, containing 1.65% cellulose, 0.16% pectin, 0.23% pectic acid, and 0.04% protopectin [15].

Free radicals are highly reactive molecular species that have an unpaired electron. Oxygen free radicals are particularly important in biological tissues because metabolism depends on electron transfer, oxidation/reduction reactions, and molecular oxygen. Therefore, their activity is a normal feature of both plant and animal cells, e.g., in electron transport, lipid metabolism, detoxification, and phagocytosis. In the solid state (or dry tissue), the life of free radicals can be much longer and range from several days to several months or even years. In nature, they can be formed during the process of photosynthesis [16]. Free radicals can initiate harmful reactions inside cells, and their action is strictly controlled. Cells are equipped with antioxidants that ensure the removal of all free radicals generated during metabolic processes. Unfortunately, such control is compromised if tissues undergo pathological disease, severe stress, or physical damage [17,18]. Products of plant origin provide unique ingredients necessary for many metabolic reactions occurring in the human body.

The antioxidant activities of various substances of natural origin, including herbal preparations, have been remarked in countless experiments using many methods and under various conditions. Unfortunately, there is often a lack of correlation between results obtained for the same material using different methods, and even between results obtained for the same samples using the same method in different research laboratories [19]. Chemical methods for determining antioxidant capacity are based on measuring the effects of antioxidants on the rate of oxidation processes occurring in the sample (ORAC and TRAP), reduction of metal ions, e.g., iron (FRAP) or copper (CUPRAC), synthetic capture ability radical (ABTS, DPPH), or measuring the amount of lipid oxidation products or LDL fraction [20]. The most frequently used radical to determine the antioxidant properties of the filtrate is DPPH, while Glv radicals are rarely used.

The aim of the present work was to determine the anti-radical effect of infusions of *Sambucus nigra* fruits or flowers using the reaction with DPPH radicals (DPPH·) and galvinoxyl radicals (Glv·). We also propose models describing the decay plots of the above radicals in the presence of the evaluated infusions.

## 2. Materials and Methods

**Figure 1:**
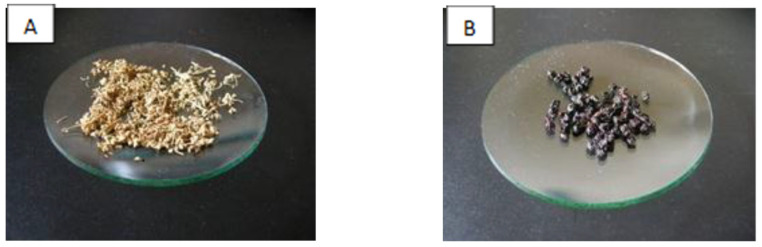
Elderberry flowers (**A**) and fruits (**B**).

The following reagents were used during the research: ethyl alcohol (CHEMPUR, Piekary Śląskie, Poland), DPPH·-C_18_H_12_N_5_O_6_ (Sigma Aldrich, St. Louis, MO, USA), Glv·-C_29_H_41_O_2_ (Sigma Aldrich, St. Louis, MO, USA), and elderberry flowers and fruits from commercial samples (Figure 1A,B).

### 2.1. Preparation of Samples for Testing

Infusions were prepared by diluting the starting solution according to Table 1.

### 2.2. Characterization of Dry Elderberry Samples

#### 2.2.1. Elemental Analysis

Elemental analysis of C, H, and N (CHN) was performed on a Carlo Erba Instruments (Thermo Scientific, Waltham, MA, USA) NA 1500 Series 2 Nitrogen/Carbon/Sulfur Analyzer. The determination of CHN involved high-temperature combustion of samples in tin pots. In the first stage, the sample was burned in the presence of catalysts at temperatures above 1000 °C. The resulting gas mixture was sent to the second stage, in which nitrogen oxides were reduced at a temperature of 650 °C and chromatographic separation took place, thereby quantitatively determining nitrogen products, carbon dioxide, and water.

#### 2.2.2. Determination of Antioxidant Properties

The infusions were obtained freshly after weighing 1 g of dried *S. nigra* flower or fruit on an analytical balance and then brewed for 8 min in a volume of 100 mL of distilled water [21]. After filtering through a filter into 100 mL volumetric flasks, the solution was allowed to cool, then appropriate dilutions were prepared from it: 1.0 × 10^−3^, 1.5 × 10^−3^, 2.0 × 10^−3^, 2.5 × 10^−3^ g·mL^−1^, according to Table 1.

The anti-free radical properties of the obtained elderberry infusions were determined in the reaction with the DPPH radical according to the modified Brand-Williams method [22] or with the galvinoxyl radical [23,24,25]. These methods enabled the observation of radical quenching initiated in the presence of an antioxidant contained in the tested infusion. The equation of the calibration curve for DPPH radical was y = 8.6885x + 0.0315 (R^2^ = 0.9994), whereas for galvinoxyl radical it was y = 18.465x + 0.0471 (R^2^ = 0.9972). The equations of the obtained standard curves were used for further measurements with initial absorbance (A_0_) of 1.1219 for DPPH and of 1.1720 for galvinoxyl.

UV–vis spectra were performed using a PG Instruments UV–Vis T60 spectrophotometer (Alab, Warsaw, Poland) coupled to a computer. The decrease in absorbance was measured at a wavelength of 517 nm for DPPH and 428 nm for galvinoxyl. The results were recorded for 1000 s at an interval of 1 s. The control sample was mixture of 0.5 mL of the tested dilution of the infusion with 3.0 mL of ethanol. The tested material was included in the control sample to ensure proper readings of absorbance in the colored samples.

The following formula was used to calculate the percentage of reacted DPPH· or Glv·:

(1) $$R\%=\frac{A_{t}}{A_{0}}\;\times \;100\%$$

where: *R*%—% remaining, *A*_0_—absorbance DPPH· or Glv· at time 0, and *A_t_*—absorbance DPPH· or Glv· at time t after mixing with elderberry extract.

#### 2.2.3. Radicals Used in Research

The DPPH· molecule was a stable π radical in which the molecular orbital contained a single electron. Its stability was primarily due to steric crowding around the divalent N atom [26,27]. The single electron of the nitrogen atom in DPPH· was reduced to the corresponding hydrazine by taking a hydrogen atom from the antioxidants [28]. Galvinoxyl is a free organic radical of outstanding chemical stability, and its solutions are known to be stable even in the presence of oxygen [29]. This stability is due to both steric hindrance induced by the bulky *tert*-butyl groups and efficient delocalization of the unpaired electron throughout the conjugated system. The reaction mechanism of DPPH· or Glv· with an antioxidant can proceed as follows [30]:

DPPH ×(or GLV·)+AH →DPPH−H (GLV−H)+A·

where: AH = antioxidant radical scavenger and A·=antioxidant radical.

### 2.3. Calculation

Simulations using mathematical equations were performed using Origin 6 and Statistica 13 [31]. The details of the applied equations are provided in the First-order kinetics rate constant of radical decay and Evaluation of the radical decay kinetics as second-order processes parts of the Section 3.

## 3. Results and Discussion

### 3.1. Elemental Analysis of CHN

The flower contained 43.75% carbon, 6.32% hydrogen, and 3.37% nitrogen, whereas the fruit included 49.27% carbon, 6.95% hydrogen, and 1.19% nitrogen. The H/C ratio was nearly equal for flowers and fruits, while the N/C ratio was larger for flowers than for fruits. Environmentally available N and C can act as signals to regulate nutrient absorption, assimilation, photosynthesis, and eventually plant growth [32]. The metabolic routes of carbon and nitrogen are closely related in various living organisms, e.g., N assimilation depends on the availability of the carbon skeleton for biosynthesis. Therefore, limitation or oversupply of one element strongly affects the metabolism of the other [33]. In the present observations, no variability was observed in the H/C ratio, which may confirm the similar properties of the antioxidants present in fruits and flowers in the terms of their molecular aromaticity.

### 3.2. Determination of the Antioxidant Properties of Elderberry Flower and Fruit Infusions

#### 3.2.1. Scavenging Effect on DPPH· and Glv· Radicals

**Figure 2:**
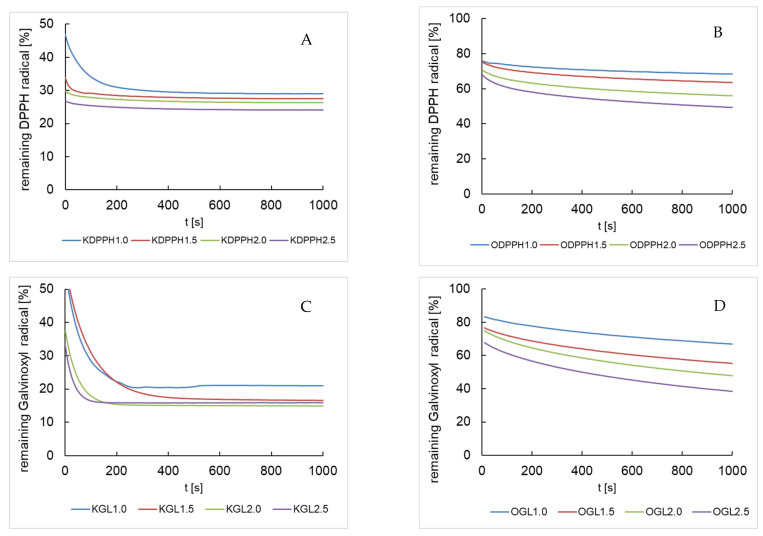
DPPH radical concentration over time after mixing with flower (**A**) or fruit (**B**) elderberry extract in various volumes and galvinoxyl radical concentration [%] over time after mixing with flower (**C**) or fruit (**D**) elderberry extract in various volumes. The acronyms are provided in Table 1.

The extinction rate of the DPPH· radical decreased with time in reaction with the elderberry flower or fruit extract components, as depicted in Figure 2A,B. The radical concentration decreased rapidly within the first few seconds and depended on the concentration of the used extract. In the elderflower samples, radical quenching already exceeded 50% at the onset of the evaluation (Figure 2A). A similar dependence of the disappearance of the DPPH radical over time was only observed in the case of fruit for the ODPPH2.5 sample, which contained the highest level of fruit extract (Figure 2B). The samples obtained from fruit failed to achieve reaction equilibrium after 1000 s, conforming the slower reaction compared to samples from flower. Our comparison of quenching by the two radicals in samples from *Sambucus nigra* flowers revealed that 30% of DPPH· remained in the sample, whereas only 20% of Glv· remained. In the case of fruit, about 50% of the DPPH· and 40% of the Glv· remained after 1000 s.

Depending on the type of substituents in the aromatic ring, molecules may react differently [34,35,36]. Unlike the chemical composition of elderberry fruits, which are especially rich in anthocyanins, the flowers do not contain pigments from this group, but are rich in flavonoids [37]. Each type of test adds a different view to the observed overall picture of potential antioxidant activity. The phenoxyl radical obtained from polyphenol in reaction with galvinoxyl can be stabilized by hydrogen bonding with the neighboring phenolic hydroxyl and the aromatic ring [38]. This stabilization promotes the reaction of polyphenols with higher number of galvinoxyl radicals, resulting in stoichiometry greater than 1:1, which can lead to higher reaction efficiency.

#### 3.2.2. Evaluation of the Radical Decay Kinetics as Parallel Processes

According to Figure 2A,B the concentration of absorbed radicals decreased over time in the presence of the extracts. The results of preliminary studies have already shown that the investigated process is complex in kinetic terms. However, a closer analysis of the experimental data indicates that in the initial period of the reaction in the evaluated systems the experimental results can be described with a good approximation by the first-order kinetic equation. This is a reaction that proceeds at a rate that depends linearly on one selected concentration of the reactant, which can be represented by the following equation:

(2) $$y=Ae^{-kt}+y_{0}$$

where: y—absorbance after time *t*, $y_{0}—$remained absorbance (absorbance after infinite time), *A*—amplitude, i.e., initial absorbance at time 0 (the difference between the absorbance at time zero and absorbance in an infinitely long time), *t*—time [s], and *k*—rate constant [s^−1^].

Respective simulations were performed using the equations for first-, second-, and third-order reactions, expressed in the applied program as equations of two or three parallel first-order reactions for the second- and third-order kinetics [39,40]:

(3) $$y=A_{r\left(1\right)}e^{\frac{-t}{t_{1}}}+y_{0}$$

(4) $$y=A_{r\left(1\right)}e^{\frac{-t}{t_{1}}}+A_{r\left(2\right)}e^{\frac{-t}{t_{2}}}+y_{0}$$

(5) $$y=A_{r\left(1\right)}e^{\frac{-t}{t_{1}}}+A_{r\left(2\right)}e^{\frac{-t}{t_{2}}}+A_{r\left(3\right)}e^{\frac{-t}{t_{3}}}+y_{0}$$

where: y—absorbance after time t, $y_{0}$—remained absorbance (absorbance after infinite time), *A_r_*_(1)_, *A_r_*_(2)_, *A_r_*_(3)_—amplitude (the difference between the absorbance at time zero and absorbance in an infinitely long time), *t*—time [s], and 1/*t*_1_, 1/*t*_2_, 1/*t*_3_—rate constants [s^−1^].

The results of the analysis of nonlinear fitting of the curve to the measurement points obtained by the least squares method are presented in Table S1 for the flower and in Table S2 for the fruit. According to Equations (3)–(5), it would appear that DPPH· and Glv· react in parallel with one, two, and three different compounds and that the rate is proportional to the concentration of only one reactant. In the presented case, this is the radical concentration. The assumption of the model is that the concentration of antioxidants does not change over time. Comparing the obtained rate constants, they are higher when using flower infusions (Table S1) than when using fruit infusions (Table S2). Taking into account R^2^ and the data obtained in the case of DPPH· decay in the flower infusions, a model of two parallel primary reactions can be used (Table S1, FO2). The introduction of the model with three parallel reactions (FO3) does not improve the fit of the curve to the obtained data. In the case of the reaction with Glv·, the obtained results are best described by the first-order reaction model (FO1). The R^2^ parameter obtained for the FO1 model of the DPPH· and Glv· radical quenching reaction by fruit infusions is lower than for FO2. Thus, it may be assumed that the radical decay is well described as two parallel reactions of the first order (FO2). The third-order model (FO3) did not provide additional information on the specific course of evaluated reactions.

#### 3.2.3. Evaluation of the Radical Decay Kinetics as Second-Order Processes

The second-order kinetics model was proposed by our team on the basis of the equation obtained after solving the second-order kinetics differential equation for different initial concentrations of DPPH· or Glv· and AH. The assumptions included the radical ($A_{r}$) and antioxidant ($A_{AH}$) absorbance change over time (t) when products (P) appear.

AH+r→P

(6) $$A_{r}=A_{r\left(0\right)}\frac{A_{AH\left(0\right)}-A_{r\left(0\right)}}{e^{\left(-ktA_{AH\left(0\right)}-ktA_{r\left(0\right)}\right)}A_{AH\left(0\right)}-A_{r\left(0\right)}}=A_{r\left(0\right)}\frac{A_{r\left(0\right)}-A_{AH\left(0\right)}}{A_{r\left(0\right)}-A_{AH\left(0\right)}e^{\left(-ktA_{r\left(0\right)}-A_{AH\left(0\right)}\right)}}$$

The differential equation was solved for initial conditions, where $A_{AH\left(0\right)}$ and $A_{r\left(0\right)}$ are the respective initial concentrations of $A_{AH}$ and $A_{r}$ in dm^3^mol^−1^s^−1^ at time 0, while $A_{AH}$ and $A_{r}$ are the concentrations after time t in seconds. The equation enables quantification of both antioxidants and radicals in respective time intervals. The compliance of the obtained results with the proposed model is discussed in terms of the parameters presented in Table S3. The resulting adherence of the experimental data with the considered second-order kinetics was statistically significant. The probability that R^2^ is zero (χ^2^ *p*-value) was zero in all cases, with accuracy to the fifth decimal position. The probability calculated above confirms the accuracy of the description of the radical quenching reaction in *Sambucus nigra* infusions. According to the obtained rate constants (k), the quenching reactions of both radicals, DPPH and Glv, were faster in the infusions obtained on the basis of *Sambucus nigra* flowers, whereas they were lower in the fruit infusions. In the case of the DPPH·, the difference was by one order of magnitude, while in the case of the Glv· it was by three orders of magnitude. When comparing the reactions of flower infusions with various radicals, the obtained rate constants in the reaction with the Glv· were higher than with the DPPH radical, while in the case of fruits it was the opposite. The rate constants should be assumed to be independent of the concentration of the reactants. In our results, they differ from each other at extreme concentrations. Higher concentrations can lead to more collisions and a greater likelihood of successful reactions. In these cases, a different kinetics model would have to be used. For comparison, extracts from six species of medicinal plants with a long history of ethnopharmacological applications (*Chelidonium majas* L., *Myrtus communis* L., *Hamamelis virginiana* L., *Juniperus communis* L., *Alchemilla vulgaris*, and *Ilex paraguariensi*) showed the following second-order reaction rate constant values: 3.25 ± 0.08, 4.3 ± 0.3, 3.72 ± 0.13, 5.85 ± 0.18, 8.8 ± 0.2, 22.9 ± 0.6 × 10^−4^ dm^3^·mol^−1^·s^−1^, all of which are much lower than *Sambucus nigra* [41].

Based on Equation (6), it is possibility to calculate the simulated initial absorbance of radical ($A_{r\left(0\right)}$) and the estimated theoretical absorbance of antioxidant ($A_{AH\left(0\right)}$), as presented in Table S4. The $A_{AH\left(0\right)}$ factor reflects the initial level of the antioxidant. However, due to the variable structures and molecular masses of different antioxidants, this may be considered as an approximation. The above equation can be further applied to calculate the levels of antioxidants revealed in the decay process of natural sources of radicals. Both single- and multicomponent antioxidant systems can be evaluated by this method. According to the above-mentioned approximative specific of the $A_{AH\left(0\right)}$ factor, in multicomponent systems only the collective amount of the antioxidants can be estimated.

## 4. Conclusions

Both free radicals and antioxidants occur in the cellulose matrices of dried *Sambucus nigra* and its infusions. Infusions obtained from flowers quenched radicals faster than fruit infusions. The most appropriate kinetic model was identified as the second-order kinetics. The equation enables quantification of both antioxidant and radical in the respective time intervals. When compared in the group of elderberry flower infusions, the rate constants obtained for the quenching reaction of the Glv radical were larger than in the case of the DPPH radical. In the case of the fruit infusions, the opposite dependency was observed and DPPH decayed faster. The applied second-order kinetics equation can enable estimation of antioxidant levels in natural sources of radicals, as demonstrated on infusions of elderberry fruit and flower.

## Figures and Tables

**Table 1:** Scheme of dilution of the starting solution of infusions of *S. nigra* flowers and fruits.

Original Infusions Composition		Evaluated Dilutions Composition				Assessed Samples Composition					
Acronym	Composition	Acronym	Original Infusion of Flos Sambuci [mL]	Original Infusion of Fructus Sambuci [mL]	Water [mL]	Acronym	Dilution [mL]	DPPH· Solution [mL]	Acronym of Assessed Sample	Dilution [mL]	GLv· Solution [mL]
K	Flos Sambuci: 1 g Water: 100 mL	K1.0	1.0	-	9.0	KDPPH1.0	0.5	3.0	KGL1.0	0.5	3.0
		K1.5	1.5	-	8.5	KDPPH1.5	0.5	3.0	KGL1.5	0.5	3.0
		K2.0	2.0	-	8.0	KDPPH2.0	0.5	3.0	KGL2.0	0.5	3.0
		K2.5	2.5	-	7.5	KDPPH2.5	0.5	3.0	KGL2.5	0.5	3.0
O	Fructus Sambuci: 1 g Water: 100 mL	O1.0	-	1.0	9.0	ODPPH1.0	0.5	3.0	OGL1.0	0.5	3.0
		O1.5	-	1.5	8.5	ODPPH1.5	0.5	3.0	OGL1.5	0.5	3.0
		O2.0	-	2.0	8.0	ODPPH2.0	0.5	3.0	OGL2.0	0.5	3.0
		O2.5	-	2.5	7.5	ODPPH2.5	0.5	3.0	OGL2.5	0.5	3.0

K—flower, O—fruit.

## Data Availability

The data presented in the manuscript are available from the Department of Physical Chemistry and Biophysics, Wroclaw Medical University.

## Supplementary Materials

The following supporting information can be downloaded at: https://www.mdpi.com/article/10.3390/antiox13070804/s1, Table S1: Kinetic and statistical parameters for infusions obtained from elderberry flowers: χ2 *p*-value (probability that R-square is zero), remained absorbance (y_0 – absorbance after infinite time), amplitude: the difference between the absorbance at time zero and absorbance in an infinitely long time (A1, A2, A3), time at 1/e of initial absorbance (t1, t2, t3), rate constants (1/t1, 1/t2, 1/t3), and determination coefficients (R2) obtained for the application of first order, second order or third order equations according to Equations (3)–(5). Details and acronyms in the text and Table 1. Table S2. Kinetic and statistical parameters for infusions obtained from elderberry fruits: χ2 *p*-value (probability that R-square is zero), remained absorbance (y_0 – absorbance after infinite time), amplitude: the difference between the absorbance at time zero and absorbance in an infinitely long time (A1, A2, A3), time at 1/e of initial absorbance (t1, t2, t3), rate constants (1/t1, 1/t2, 1/t3), and determination coefficients (R2) obtained for the application of first order model (FO1), two parallel reactions (FO2) or three parallel reactions (FO3) according to Equations (3)–(5). Details and acronyms are in the text and Table 1. Table S3. Kinetic and statistical parameters for infusions obtained from elderberry flowers and fruits: χ2 *p*-value (probability that R-square is zero), rate constants (k), and determination coefficients (R2) obtained as result of the application of the second order kinetics equation, acc. to Equation (6). Details and acronyms are in the text and Table 1. The second-order kinetic differential equation was solved for various initial concentrations of DPPH·or Glv·and AH taking into account that both radical and antioxidant concentrations change with time. Table S4. Estimated initial absorbances: of the radical (A_r) and of the antioxidants (A_AH) in infusions of elderberry fruit (ODPPH, OGL) and elderberry flower (KDPPH, KGL), calculated according to the Equation (6).
