# Supplementary material for: Evaluation of Decay Kinetics of Black Elderberry Antioxidants from Fruits and Flowers
Source: Antioxidants (Basel). 2024 Jul 2;13(7):804. doi: 10.3390/antiox13070804 (PMC11274222; doi:10.3390/antiox13070804)
Supplement: Supplementary file 1 [file antioxidants-13-00804-s001.zip › antioxidants-3048936-supplementary.pdf]

Table 1s. Kinetic and statistical parameters for infusions obtained from elderberry flowers: χ^2 p-value (probability that R-square is zero), remained absorbance (y_0 – absorbance after infinite time), amplitude: the difference between the absorbance at time zero and absorbance in an infinitely long time (A_1, A_2, A_3), time at 1/e of initial absorbance (t_1, t_2, t_3), rate constants ($1/t_1$, $1/t_2$, $1/t_3$), and determination coefficients (R^2) obtained for the application of first order, second order or third order equations according to Eq. 3-5. Details and acronyms in the text and Table 1.

Kinetic Model	Kinetic Parametres	KDPPH1	KDPPH1.5	KDPPH2	KDPPH2.5	KGL1	KGL1.5	KGL2	KGL2.5
FO1	χ^2 p-value	0.000005	0.000006	0.000000	0.000000	0.000010	0.000006	0.000005	0.000005
	y_0	0.32873±0.00006	0.31127±0.00008	0.29663±0.00003	0.31137±0.00002	0.19081±0.00017	0.24367±0.00013	0.1727± 0.0001	0.18385±0.00011
	A_1	0.18489±0.00035	0.04229±0.00033	0.03148±0.00009	0.02601±0.00005	0.4682±0.0017	0.4144±0.0016	0.26559±0.0016	0.2030±0.0018
	t_1 [s]	85.88±0.26	121.0±1.6	187.7±1.1	197.01±0.87	102.03±0.58	63.71±0.38	50.77±0.47	31.96±0.47
	$1/t_1$ [s^{-1}]	0.01164±0.00007	0.00827±0.00022	0.00533±0.00006	0.00508±0.00004	0.00980±0.00011	0.01570±0.00019	0.01970±0.00036	0.03130±0.00092
	R^2	0.99559	0.92542	0.98788	0.99299	0.99711	0.9969	0.99241	0.9816
FO2	χ^2 p-value	0.000000	0.000000	0.000000	0.000000	0.000000	0.000006	0.000005	0.000006
	y_0	0.32605±0.00003	0.30946±0.00002	0.29597±0.00001	0.31094±0.00000	0.18655±0.00020	0.24367±0.00013	0.1727±0.00012	0.18385±0.00011
	A_1	0.0473±0.0007	0.02761±0.00005	0.02752±0.00004	0.00783±0.00006	0.46256±0.00053	0.20719±--	0.1328±--	0.10149±--
	t_1 [s]	202.4±2.0	230.28±0.74	231.79±0.57	20.83±0.30	94.40±0.22	64±20000	51±120000	1.95971±--
	$1/t_1$ [s^{-1}]	0.00494±0.00010	0.00434±0.00003	0.00431±0.00002	0.04802±0.00138	0.01059±0.00005	0.00000±0.00010	0.00000±0.00002	0.51028±0.00000
	A_2	0.14968±0.00065	0.04373±0.00011	0.01278±0.00007	0.02347±0.00003	0.01699±0.00029	0.20719±--	0.1328±--	0.10149±--
	t_2 [s]	60.55±0.23	14.898±0.069	19.05±0.20	231.22±0.57	1205±56	64±20000	51±120000	31.95971±--
	$1/t_2$ [s^{-1}]	0.01652±0.00013	0.06712±0.00062	0.05250±0.00110	0.00432±0.00002	0.00083±0.00008	0.00000±0.00010	0.00000±0.00002	0.03129±0.00000
	R^2	0.99980	0.99905	0.99931	0.99931	0.99981	0.99690	0.99241	0.98160
FO3	χ^2 p-value	0.000000	0.000000	0.000000	0.000000	0.000000	0.000000	0.000000	0.000005
	y_0	0.32605±0.00004	0.30944±0.00002	0.29597±0.00003	0.31094±0.00003	0.18568±0.00014	0.24367±0.00013	0.1618±3734.74897	0.18385±0.00012
	A_1	0.07485±1094.15976	0.00313±0.00093	0.01376±--	0.01174±144.39873	0.02393±0.00082	0.13812±72996.32433	0.1338±1621.493	0.06766±26642.94732
	t_1	60.55451±1336349.22335	4.44734±1.30123	231.79277±43381.20014	231.21920±335172.10288	15.52797±0.82679	63.70465±--	47.69309±0.00073	31.95974±59743740.40455
	$1/t_1$ [s^{-1}]	0.00000±0.00000	0.24590±0.14390	0.00000±0.00005	0.00000±0.00001	0.06458±0.00688	0.01570±0.00000	0.02097±0.00073	0.00000±0.00000
	A_2	0.07483±1094.15885	0.04155±0.00095	0.01376±--	0.01174±144.39874	0.01549±0.00011	0.1381±47152.9366	0.01569±361.07724	0.06766±4128.34898
	t_2	0.32605±0.00004	15.62649±0.24137	43380.4455±--	231.221±335172.457	1549.993±43.692	63.70465±--	4960.03348±3734.74897	31.95967±1538636.26304
	$1/t_2$ [s^{-1}]	3.06701±0.00075	0.06401±0.00198	0.00030±0.00000	0.00000±0.00001	0.00065±0.00004	0.01570±0.00000	0.00047±0.00070	0.00000±0.00000
	A_3	0.04730±0.00156	0.02747±0.00006	0.01278±0.00009	0.00783±0.00008	0.44999±0.00074	0.1381±62340.9491	0.12994±1672.60815	0.06766±47188.31536
	t_3	202.38839±3.46604	231.85530±0.82226	19.04730±0.25704	20.82764±0.38849	98.11793±0.18635	63.7059±56693.6827	47.80933±3734.74897	31.95974±58365916.90975
	$1/t_3$ [s^{-1}]	0.00494±0.00017	0.00431±0.00003	0.05251±0.00142	0.04803±0.00179	0.01019±0.00004	0.00000±0.00004	0.00000±0.00054	0.00000±0.00000
	R^2	0.99980	0.99907	0.99931	0.99931	0.99997	0.99690	0.99991	0.98160

FO1 - first order model. FO2 - two parallel primary reactions. FO3 - three parallel primary reactions

Table 2s. Kinetic and statistical parameters for infusions obtained from elderberry fruits: χ^2 p-value (probability that R-square is zero), remained absorbance (y_0 – absorbance after infinite time), amplitude: the difference between the absorbance at time zero and absorbance in an infinitely long time (A1, A2, A3), time at 1/e of initial absorbance (t1, t2, t3), rate constants (1/t1, 1/t2, 1/t3), and determination coefficients (R2) obtained for the application of first order model (FO1), two parallel reactions (FO2) or three parallel reactions (FO3) according to Eq. 3-5. Details and acronyms are in the text and Table 1.

Kinetic Model	Kinetic Parametres	ODPPH1	ODPPH1.5	ODPPH2	ODPPH2.5	OGL1	OGL1.5	OGL2	OGL2.5
FO1	χ^2 p-value	0.00006	0.00002	0.00003	0.00005	0.00003	0.00006	0.00008	0.00008
	y_0	0.72735±0.00018	0.66284±0.00026	0.5670±0.0003	0.47671±0.00041	0.58502±0.00197	0.41665±0.00242	0.33015±0.00206	0.23521 ±0.00166
	A_1	0.11080±0.00016	0.15221±0.00025	0.18988±0.0003	0.2332±0.0004	0.36319±0.00162	0.43863±0.00203	0.4953±0.00194	0.50735±0.0018
	t_1	1008.94691±4.55694	911.02320±4.71130	874.03506±4.35946	885.33042±4.76685	1706.17033±22.65177	1594.34779±22.74022	1326.03848±16.45694	1171.78065 ±12.64731
	$1/t_1$ [s^{-1}]	0.00099±0.00001	0.00110±0.00001	0.00114±0.00001	0.00113±0.00001	0.00059±0.00002	0.00063±0.00002	0.00075±0.00002	0.00085±0.00002
	R^2	0.99199	0.98785	0.98801	0.98630	0.99616	0.99506	0.99506	0.99556
FO2	χ^2 p-value	0.00000	0.000000	0.000000	0.000002	0.000000	0.000000	0.000002	0.000002
	y_0	0.70628±0.00025	0.63734±0.00022	0.5389±0.0002	0.44242±0.00026	0.49542±0.00254	0.3261±0.0021	0.26964±0.00117	0.19047±0.00079
	A_1	0.1111±0.0001	0.05562±0.00014	0.18098±0.00011	0.0893±0.0002	0.09011±0.00114	0.11319±0.00108	0.13374±0.00119	0.14008±0.00127
	t_1	1786.2±9.4	125.45±0.65	1478.6±4.5	106.47515±0.49176	348.23740±5.32986	290.10418±3.95657	257.09037±3.32556	255.99517±3.16113
	$1/t_1$ [s^{-1}]	0.00056±0.00001	0.00797±0.00008	0.00068±0.00000	0.00094±0.00942	0.00287±0.00009	0.00345±0.00013	0.00389±0.00010	0.00391±0.00010
	A_2	0.03392±0.00014	0.14820±0.00012	0.06997±0.00016	0.22464±0.00014	0.38688±0.00142	0.45241±0.00109	0.46659±0.00055	0.45547±0.00077
	t_2	165.61498±1.14862	1587.5±5.7	122.35±0.57	1474.72601±4.23427	3196.34722±45.19769	2815.31154±29.76211	2071.69601±15.13671	1738.5785±10.60746
	$1/t_2$ [s^{-1}]	0.00604±0.00008	0.00063±0.00000	0.00817±0.00008	0.00068±0.00000	0.00031±0.00001	0.00036±0.00001	0.00048±0.00001	0.00058±0.00001
	R^2	0.99954	0.99958	0.99966	0.99962	0.99993	0.99992	0.99993	0.99994
FO3	χ^2 p-value	0.00000	0.00000	0.00000	0.00000	0.00000	0.00000	0.00000	0.00000
	y_0	0.70628±0.00043	0.61891±0.00051	0.52379±0.00031	0.41315±0.00046	0.44942±1.26876	0.31058±0.49377	0.21165±0.16184	0.14412±0.00511
	A_1	0.03392±0.00015	0.15167±0.00022	0.1806±0.0001	0.07064±0.00021	0.2111±3.6924	0.23395±9.61156	0.11461±0.00863	0.20264±0.00911
	t_1	165.61491±1.17265	2184.93333±17.17147	1875.0388±8.3841	269.72238±1.89043	2271.06868±12985.95072	2195.88741±20434.89013	223.50212±10.09132	638.75189±26.87113
	$1/t_1$ [s^{-1}]	0.00000±-0.00012	0.00046±0.00001	0.00053±0.00000	0.00000±0.00015	0.00001±-0.00010	0.00010±0.00010	0.00448±0.00040	0.00157±0.00013
	A_2	0.05482±--	0.0491±0.0002	0.03266±0.00024	0.05446±0.00023	0.08452±0.01783	0.10976±0.01448	0.27759±0.21951	0.3788±0.0061
	t_2	1786.1991±1269690	255.9503±2.6085	41.44219±0.42939	48.04732±0.26965	331.66231±31.85667	281.89595±19.22736	4362.86833±8810.85603	2724.18588±130.97158
	$1/t_2$ [s^{-1}]	0.00000±0.00000	0.00391±0.00008	0.02413±0.00050	0.02081±0.00023	0.00304±0.00058	0.00356±0.00049	0.00007±0.00030	0.00037±0.00004
	A_3	0.05629±--	0.02756±0.00024	0.06041±0.00018	0.22677±0.00016	0.22764±2.44362	0.2377±9.1329	0.26756±0.37197	0.06584±0.00222
	t_3	1786.2010±12365723	44.33617±0.54719	230.13991±1.71505	2101.1826±210.44523	6266.9969±127181.55	3975.9571±74362.28050	1338.2375±805.22068	131.362±3.57041
	$1/t_3$[s^{-1}]	0.00000±0.00000	0.02256±0.00056	0.00435±0.00006	0.00048±0.00010	0.00000±0.00002	0.00000±0.00003	0.00117±0.00141	0.00762±0.00391
	R^2	0.99954	0.99989	0.99993	0.99996	0.99994	0.99993	0.99995	0.99999

O1 - first order model. O2 - two parallel primary reactions. O3 - three parallel primary reactions

Table 3s. Kinetic and statistical parameters for infusions obtained from elderberry flowers and fruits: χ^2 p-value (probability that R-square is zero), rate constants (k), and determination coefficients (R^2) obtained as result of the application of the second order kinetics equation, acc. to Eq. 6. Details and acronyms are in the text and Table 1. The second-order kinetic differential equation was solved for various initial concentrations of DPPH· or Glv· and AH taking into account that both radical and antioxidant concentrations change with time.

Kinetic parameters	KDPPH1.0	KDPPH1.5	KDPPH2.0	KDPPH2.5	KGL1.0	KGL1.5	KGL2.0	KGL2.5
χ^2 p-value	0.00000	0.00000	0.00000	0.00000	0.00000	0.00000	0.0000	0.0000
k [$dm^3mol^{-1}s^{-1}$]	0.04038±0.00100	0.1663±0.0015	0.11129±0.00378	0.10677±0.00331	0.1960±0.0012	0.3139±0.0018	0.3939±0.0038	0.6407±0.0026
R^2	0.99959	0.9992	0.994787	0.996443	0.997102	0.996902	0.992414	0.99986

Kinetic Parametres	ODPPH1.0	ODPPH1.5	ODPPH2.0	ODPPH2.5	OGL1.0	OGL1.5	OGL2.0	OGL2.5
χ^2 p-value	0.00000	0.00000	0.00000	0.00000	0.00000	0.00000	0.00000	0.00000
k [$dm^3mol^{-1}s^{-1}$]	0.05915±0.00268	0.01928±0.00117	0.01853±0.00113	0.02405±0.00073	0.00063±0.00001	0.000750±0.000011	0.000967±0.000010	0.001244±0.000009
R^2	0.982063	0.996463	0.997998	0.99892	0.997661	0.997422	0.998241	0.99912

Table 4s. Estimated initial absorbances: of the radical (A_r) and of the antioxidants (A_{AH}) in infusions of elderberry fruit (ODPPH, OGL) and elderberry flower (KDPPH, KGL), calculated according to the Eq. 6.

Acronym	A_r	A_{AH}	Acronym	A_r	A_{AH}
KDPPH1.0	0,5635±0,0008	0,1938±0,0018	KGL1.0	0.191±0.000	3.06905±0.04278
KDPPH1.5	0.4258±0.0007	0.0948±0.0006	KGL1.5	0.244±0.000	8.41777±0.17029
KDPPH2.0	0.3450±0.0004	0.0291±0.0002	KGL2.0	0.173±0.000	11.62193±0.46878
KDPPH2.5	0.308±0.000	0.0198±0.0001	KGL2.5	0.185±0.000	94.41214±2.44825
ODPPH1.0	0.8701±0.0009	0.03328±0.00070	OGL1.0	0.956±0.001	0.4044±0.0019
ODPPH1.5	0.857±0.000	0.06658±0.00200	OGL1.5	0.8696±0.0013	0.5139±0.0028
ODPPH2.0	0.809±0.000	0.08856±0.00292	OGL2.0	0.8479±0.0014	0.5946±0.0022
ODPPH2.5	0.784±0.000	0.10830±0.00146	OGL2.5	0.7722±0.0011	0.6293±0.0016
